# Factors Related to Changes in CD4+ T-Cell Counts over Time in Patients Living with HIV/AIDS: A Multilevel Analysis

**DOI:** 10.1371/journal.pone.0084276

**Published:** 2014-02-05

**Authors:** Ulisses Ramos Montarroyos, Demócrito Barros Miranda-Filho, Cibele Comini César, Wayner Vieira Souza, Heloisa Ramos Lacerda, Maria de Fátima Pessoa Militão Albuquerque, Mariana Freitas Aguiar, Ricardo Arraes de Alencar Ximenes

**Affiliations:** 1 Federal University of Pernambuco, Department of Tropical Medicine, Recife, Pernambuco, Brazil; 2 State University of Pernambuco, School of Medical Sciences,Oswaldo Cruz University Hospital, Recife, Pernambuco, Brazil; 3 Departament of Statistics, Federal University of Minas Gerais, Belo Horizonte, Minas Gerais, Brazil; 4 Department of Public Health, Research Center Aggeu Magalhães, Osvaldo Cruz Fundation, Recife, Pernambuco, Brazil; University of Pittsburgh, United States of America

## Abstract

**Background:**

The measurement of CD4+ T-cell (CD4) counts is a strong predictor of progression to AIDS and a means of monitoring antiviral therapy (ART). The success or failure of controlling virus levels in untreated patients or those taking ART may be associated with treatment adherence, habits, correlated infections unrelated to HIV, cancer, immunosuppressive drugs; as well as socio-economic and psychosocial aspects and access to healthcare. The aim of the present study was to identify, using a multilevel model, the factors related to the variations of CD4 counts over time, in patients living with HIV.

**Methodology:**

A cohort study was conducted with patients living with HIV, selected from July 2007 to December 2010. Patients were monitored from records of their first CD4 count after being diagnosed with HIV. A multilevel model with 3 levels of aggregation was applied to analyze the associations of predictor variables and the behavior of CD4 over time.

**Principal Findings:**

A total of 1870 patients were enrolled. The mean number of CD4 at the beginning of the cohort was 393.1 cells/mm^3^, and there was a mean increase of 1.529 cells/mm^3^ per month. Patient's age, smoking, use of illicit drugs, hospital treatment, changing doctors and the use of ART, were factors that affected the kinetics of the CD4 count during the follow-up period.

**Conclusion/Significance:**

The results of this study indicated increased levels of CD4 over time in a cohort of patients living with HIV/AIDS and identified factors that may influence this increase and are liable to intervention.

## Introduction

The measurement of CD4+ T-cell (CD4) counts is a strong predictor of progression to Human Immunodeficiency Syndrome (AIDS), as well as a means of monitoring antiretroviral therapy (ART). Low CD4 counts are associated with a greater risk of patients living with HIV developing opportunistic infections, which may then progress to advanced diseases and death [1], [2].

Possible increases or decreases in CD4 counts are directly related to HIV replication. The use of combinations of antiretroviral drugs (ART) generally results in the suppression of virus replication and hence increased levels of CD4. The success or failure in controlling levels in untreated patients or those on antiretroviral therapy may be associated with factors related to treatment adherence, habits, other correlated infections unrelated to HIV, cancer, immunosuppressive drugs (corticosteroids and chemotherapy), as well as socio-economic and psychosocial factors and access to healthcare [1]–[3].

A number of studies have shown that lower age groups, poor education and low income were all associated with a poorer therapeutic and immune response. The most indicated mediating mechanisms amongst the socioeconomic variables and therapeutic response, are lower rates of treatment adherence [4]–[6] and difficulty in accessing healthcare [6]. There is no consensus in the literature on the relationship between factors such as smoking, alcohol and illicit drug use and their effect on the CD4 count over time. Some authors suggest that these factors may be related to adherence to antiretroviral treatment [4], [7], or as a direct agent in immunosuppression, as in the case of alcohol and illicit drug abuse [8].

The use and duration of ART are important factors in understanding the kinetics of viremia and CD4 counts. The work of Chu *et al.* [9], assuming the hypothesis that during the first 2 years after initiating ART, there is an increased CD4 count followed by a stabilization process, observed that the moment of stabilization varies according to the initial CD4 count, with significant effects on increase rates during treatment. Thus, patients whose initial CD4 counts were low presented a more rapid early rise and change points occurred earlier. However, Nicastri *et al.* [10] encountered an inverse relationship with no immune response and initially high levels of CD4. With regard to treatment, prolonged periods of therapy contributes to improved adherence [5], suggesting that with time patients become more committed to following the treatment as they begin to observe benefits. However, there is no consensus with regard to this aspect. In some studies, there is an inverse relationship between the treatment time and adherence [11].

Factors related to CD4 variations in patients living with HIV are multiple and complex. There are few longitudinal studies reported in the literature with long observation periods and prospective designs. A model, which is able to indicate independent associations related to CD4, with an appropriate methodology, would provide support for possible therapeutic interventions and, consequently, a better quality of life and survival for patients. Multilevel analysis takes into account the nested data structure (repeated measures of CD4 within subjects) and allows to examine relations between variables at different levels of the multilevel data structure. This study aimed to assess an explanatory model, using the statistical technique of multilevel models for the variations of CD4 counts over time in patients living with HIV.

## Materials and Methods

A cohort study was conducted with patients living with HIV, of both sexes, aged 18 years or older, with a minimum of two CD4 cell count, selected during the period from July 2007 to December 2010, treated at 2 referral HIV/AIDS centres: the Correia Picanço Hospital and the Oswaldo Cruz University Hospitals, responsible for the care of about 70% of patients living with HIV in the state of Pernambuco, Brazil. All selected patients were followed and data were obtained from a questionnaire periodically applied and from medical records. The study was approved by the research ethics committee at the University of Pernambuco Oswaldo Cruz University Hospital, and all participants who agreed to participate in this study signed a consent form.

To assess the variation of the CD4 cell count over time data was retrieved from medical records starting from the first CD4 cell count, performed at the time of HIV diagnosis. For those participants diagnosed before July 2007, CD4 counts were recorded retrospectively. The CD4 cell count for all patients was performed by flow cytometry using anti-CD4 antibodies labeled with fluorescent dye, at a reference laboratory. To get the information on biological and socioeconomic aspects, habits and disease history, patients were interviewed by previously trained health professionals, using standardized questionnaires. Data regarding the time-dependent variables, use and regimen of antiretroviral therapy, were collected from patient records.

Initial data analysis used descriptive statistics to characterize the study population. For the analysis of factors associated with CD4 variations over time, a multilevel modelling [10], [12] was used in order to adjust the complex data structure resulting from the measurements of CD4. Three levels were considered: the first level referred to an individual's repeated measurements of CD4 counts over time (level 1), the second level was represented by the individual to which the set of CD4 measurements belonged (level 2) and the third level was the grouping of patients per doctor (level 3).

The multilevel model was adjusted using GLLAMM (generalized linear latent and mixed models), and the estimated parameters of the model obtained by REML (restricted maximum likelihood estimation). Snedecor's F statistics was used to test the significance of the fixed effects model and the likelihood ratio obtained in REML was used to test the significance of the random effects. The assumptions of normality were tested estimating a regression model for each trajectory of each individual of the cohort and observing the distribution of the coefficients (intercept and slope). These assumptions were confirmed and a linear rate of change in CD4 counts over time was assumed, meeting the assumptions of generalized linear models (GLM).

In a first step of the statistical analysis, the effect of each independent variable on CD4 variation over time (interaction) and the differences in the mean CD4 count at the beginning of follow-up (intercept) were tested. With this first approach, the variables that presented a statistical significance (p-value) of the interaction parameter below 20% (p<0.20) were eligible for the multivariate analysis. In this phase, the model was adjusted following the stepwise method, with the inclusion of the variables in the model through order of significance in the univariate step (forward process). The exclusion criterion for the final multivariate model was a statistical significance below 10% (p<0.10). A residual analysis was performed from the final model. STATA (Data Analysis and Statistical Software) 12.0T was used for the descriptive analysis and construction of graphs, and R Project for Statistical Computing 2.14.2 was used in the application of multilevel models.

## Results

The study collected 21,601 measurements of CD4 counts, corresponding to 1,870 patients treated by 44 doctors in the two referral hospitals. The mean number of CD4 measurements per patient was 12±7 measurements, with a minimum record of 2 and a maximum of 40 repetitions.

A total of 533 patients were treated at the Oswaldo Cruz University Hospital and 1337 at the Correia Picanço Hospital, with a mean follow-up period of 6.0±3.6 years, with a minimum period of 10 days and a maximum of 20 years. The majority of patients surveyed were male (63.4%), with a mean age of 39.1 years, a minimum age of 18 and a maximum of 74 years. With regard to education, the number of years of study was between 1 and 9 years for 53.5% of the patients, and between 10 and 12 for 30.3%. The income of the majority of participants (64.4%) was below the minimum salary. With regard to their habits, 29.6% of patients living with HIV were smokers, 35.6% were drinkers and 27.6% used or had used illicit drugs. Current or past use of marijuana was reported by 26.6% of those surveyed, while the use of cocaine was reported by 9.1%, crack by 6.5% and glue by 5.8% of patients. Diabetes was reported in 4% of patients, cancer or lymphoma in 2.5% and the use of corticosteroids up to 6 months prior to the interview in 5.3% (Table 1).

**Table 1:** Characteristics of a cohort of patients living with HIV according to biological and socioeconomic factors, habits and personal history.

Characteristics	Statistics
**Patients**	1,870
**Biological**	
Sex: Male	1,186 (63.4%)
Age (mean ± sd)	39.1±9.5
**Socioeconomic**	
Schooling (years of study)	
Illiterate	95 (5.1%)
From 1 to 9 years	997 (53.5%)
From 10 to 12 years	564 (30.3%)
13 or more	208 (11.1%)
Income (in minimum wage)	
Less than 1MW	1,205 (64.4%)
From 1 to 2 MW	378 (20.2%)
2 MW or more	254 (13.6%)
No information	33 (1.8%)
**Habits**	
Smoking	553 (29.6%)
Drinking	666 (35.6%)
Illicit drug use	517 (27.6%)
Merijuana	498 (26.6%)
Cocaine	170 (9.1%)
Crack	122 (6.5%)
Glue	109 (5.8%)
**Personal history**	
Diabetes mellitus	75 (4.0%)
History of cancer	46 (2.5%)
Corticoid use	99 (5.3%)

* 6 patients had no information on schooling.

In an exploratory stage, noting the growth rate of the mean CD4 levels of patients throughout the follow-up period associated with each of the explanatory variables, it was observed that, among the biological variables, male patients presented a lower CD4 baseline and a lower growth rate, although not statistically significant (p = 0.163). According to age, patients aged over 40 years presented a lower increase of CD4 over time. The socioeconomic status of the patients, represented by the variables of education and income, did not discriminate different behaviours in the CD4 kinetics during the follow-up period (Table 2).

**Table 2:** Multilevel model of the changes in CD4 + T cells count over time for patients living with HIV/AIDS, according to biological and socioeconomic factors, habits, associated diseases, medical care and HIV related factors.

	Intercept		Time		Independent variables		Time interaction	
Variables	Coef. (SE)	p-value	Coef. (SE)	p-value	Coef. (SE)	p-value	Coef. (SE)	p-value
**Basic model**	338.6 (6.8)	0.000	3.091 (0.20)	0.000	-	-	-	-
**Biological and socioeconomic**								
Sex: male	354.4 (9.4)	0.000	3.307 (0.25)	0.000	−25.46 (9.9)	0.011	−0.334 (0.24)	0.163
Age group: >40 years	331.3 (8.5)	0.000	3.333 (0.23)	0.000	14.34 (9.6)	0.136	−0.498 (0.23)	0.030
Schooling: <9 years	336.5 (7.8)	0.000	3.083 (0.22)	0.000	5.55 (9.3)	0.553	0.013 (0.19)	0.943
income: <1 MW	334.7 (7.6)	0.000	3.032 (0.21)	0.000	11.52 (9.1)	0.206	0.109 (0.11)	0.328
**Habits**								
Smoking	331.5 (7.5)	0.000	3.264 (0.22)	0.000	24.59 (10.3)	0.017	−0.573 (0.22)	0.009
Drinking	334.2 (7.6)	0.000	3.204 (0.22)	0.000	12.97 (10.4)	0.168	−0.281 (0.17)	0.102
Ilicit drug use: Yes	333.0 (7.4)	0.000	3.245 (0.21)	0.000	18.71 (10.3)	0.069	−0.545 (0.21)	0.009
Marijuana	333.5 (7.4)	0.000	3.233 (0.21)	0.000	19.65 (10.2)	0.055	−0.507 (0.19)	0.008
Cocaine	335.7 (6.9)	0.000	3.123 (0.20)	0.000	34.81 (15.2)	0.022	−0.298 (0.22)	0.182
Crack	337.4 (6.9)	0.000	3.126 (020)	0.000	19.36 (17.5)	0.267	−0.413 (0.23)	0.078
Glue	336.6 (6.9)	0.000	3.124 (0.20)	0.000	38.00 (18.7)	0.042	−0.425 (0.24)	0.073
**Personal history**								
Diabetes mellitus	334.1 (5.1)	0.000	2.971 (0.12)	0.000	31,69 (22.6)	0.161	−0.054 (0.09)	0.528
History of cancer	339.6 (6.8)	0.000	3.087 (0.20)	0.000	−26.80 (27.5)	0.329	0.029 (0.16)	0.856
Corticoid use	337.1 (7.0)	0.000	3.131 (0.21)	0.000	0.034 (19.0)	0.998	−0.143 (0.09)	0.112
**Medical care**								
Hospital: Hospital A	351.29 (6.9)	0.000	2.626 (0.20)	0.000	−38.16 (12.2)	0.002	1.457 (0.35)	0.000
Changing doctor: Yes	339.55 (8.0)	0.000	3.384 (0.24)	0.000	−2.870 (10.4)	0.783	−0.604 (0.26)	0.019
**Use of ART**								
Use of HAART: Yes	319.75 (7.6)	0.000	1.715 (0.24)	0.000	40.58 (5.3)	0.000	1.136 (0.17)	0.000
HAART classes	319.75 (7.5)	0.000	1.813 (0.24)	0.000	-	-	-	-
2NRTIs+NNRTI	-	-	-	-	43.40 (6.5)	0.000	0.832 (0.18)	0.000
2NRTIs+PI	-	-	-	-	19.89 (9.3)	0.032	1.478 (0.21)	0.000
2NRTIs+PI/r	-	-	-	-	63.43 (9.0)	0.000	0.944 (0.20)	0.000
NRTI+NNRTI+PI	-	-	-	-	−31.48 (23.1)	0.173	1.362 (0.36)	0.000
NRTI+NNRTI+PI/r	-	-	-	-	43.98 (16.6)	0.000	0.699 (0.30)	0.021

With regard to the variables related to habits, a lower increase rate was observed in the mean levels of CD4 in patients with a history of smoking, drinking and illicit drug use. When evaluating each of the illicit drugs, all patients presented a lower increase in CD4 over time, although the difference was only statistically significant for patients who reported marijuana use (Table 2).

With regard to past medical history, patients with diabetes and cancer, presented no statistically significant difference in the behavior of CD4 over time, when compared to individuals without the diseases. Similar results were obtained when comparing those who reported using steroids during the 6-month period prior to the interview with those who had never used this medication (Table 2).

With respect to hospital care, it was observed that patients treated in one of the referral hospitals presented a statistically higher rate of CD4 increase when compared to patients treated in the other. Patients who changed doctors over the period of outpatient treatment, presented a lower increase in CD4 over time, and this difference was statistically significant (Table 2).

Patients on ART had a significantly higher mean rate of CD4 increase during the cohort. For patients on different regimens of ART, all regimens presented a significant increase in CD4 levels, when compared to patients who were not taking ART (Table 2).

**Figure 1:**
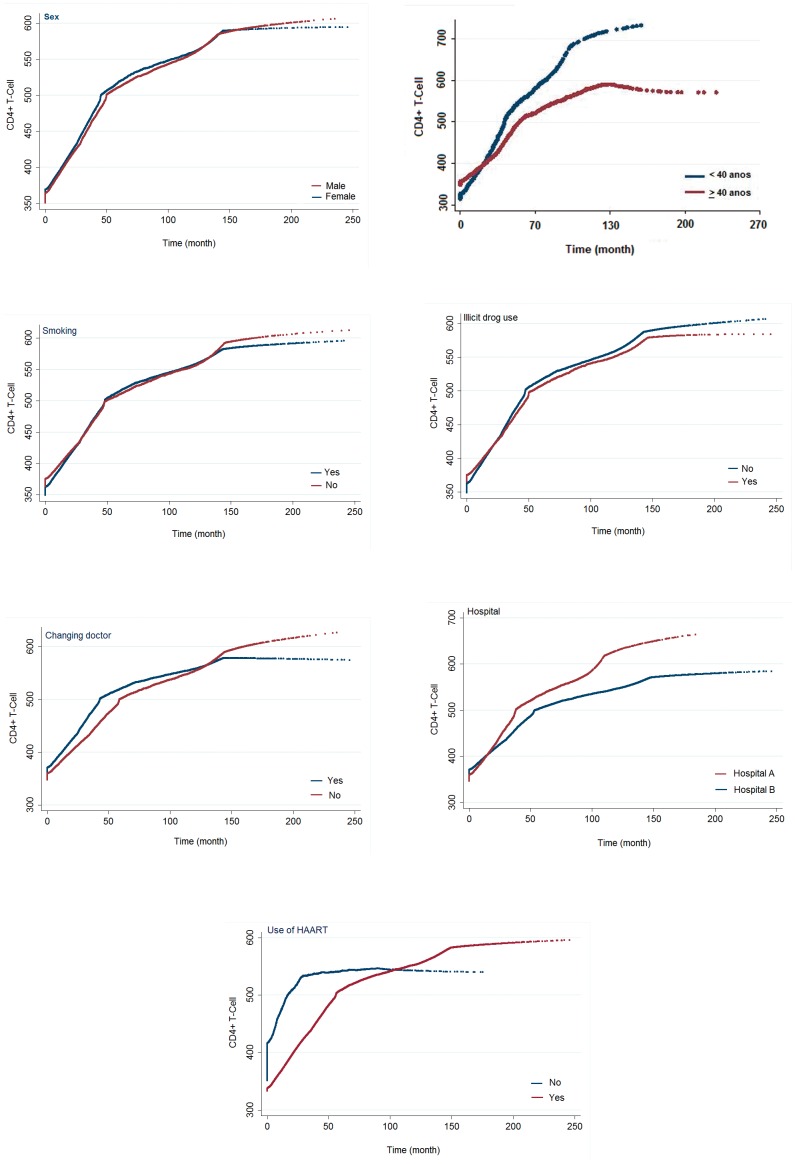
Changes in CD4 count of patients with HIV, over time, according to the variables in the final multivariate multilevel model.

Figure 1 presents the mean CD4 trajectory throughout the follow-up period of the cohort according to the explanatory variables that presented a statistically significant association with growth rates (interaction with time).

By applying the multivariate multilevel model, the estimated mean number of the CD4 count at the start of the cohort was 393.1 cells/mm^3^, with a linear increase, irrespective of the variables that make up the model, of 1.529 cells/mm^3^ per month (Table 3).

**Table 3:** Multivariate multilevel model of the changes in CD4 + T cells over time of patients living with HIV/AIDS, including separately smoking (Model 1) and illicit drug use (Model 2).

	Intercept		Time		Independent variables		Time interaction	
Variables	Coef. (SE)	p-value	Coef. (SE)	p-value	Coef. (SE)	p-value	Coef. (SE)	p-value
**Model 1**	343.3 (12.8)	0.000	2.079 (0.34)	0.000	-	-	-	-
Sex: male					−31.40 (10.4)	0.002	−0.259 (0.24)	0.274
Age group: >40 years					16.41 (9.9)	0.100	−0.536 (0.23)	0.018
Smoking					18.05 (10.7)	0.056	−0.490 (0.22)	0.024
Hospital: Hospital A					−42.50 (12.8)	0.001	1.318 (0.35)	0.000
Changing doctor: Yes					−9.41 (10.7)	0.382	−0.454 (0.25)	0.074
Use of HAART: Yes					41.12 (5.4)	0.000	1.149 (0.17)	0.000
**Model 2**	345.2 (12.5)	0.000	2.004 (0.34)	0.000	-	-	-	-
Sex: male					−33.85 (10.5)	0.001	−0.228 (0.24)	0.338
Age group: >40 years					13.18 (10.1)	0.190	−0.486 (0.23)	0.033
Ilicit drug use: Yes					26.76 (10.9)	0.003	−0.489 (0.21)	0.020
Hospital: Hospital A					−43.16 (12.8)	0.001	1.330 (0.35)	0.000
Changing doctor: Yes					−11.02 (10.7)	0.303	−0.427 (0.25)	0.093
Use of HAART: Yes					41.13 (5.4)	0.000	1.155 (0.17)	0.000

The variable sex remained in the explanatory model of CD4 kinetics for the adjustment of the association of other variables in the study, although the association was not statistically significant. Patients over 40 years of age presented a lower monthly increase of CD4, after adjustment for the other variables. Smoking presented a significant effect on the levels of CD4 throughout the cohort. (Table 3).

Two variables related to the healthcare patients received, which were the hospital and changing doctors during outpatient follow-up, remained in the explanatory model. Patients from one of the referral hospitals presented a higher rate of CD4 increase, and the fact that patients had changed doctors during the follow-up period was associated with a lower rate of CD4 increase over time, which was statistically significant (Table 3).

The use of ART remained in the explanatory model associated with a statistically significant increase of CD4 levels over the period of follow-up (Table 3).

Table 3 also presents another model considering the variable illicit drug use instead of smoking, and it was observed that patients who used any illicit drug, either at the time of the interview or in the past, had a lower rate of CD4 increase over time.

## Discussion

From a prospective cohort study, using a statistical method of multilevel modeling, it was observed that patient age, the habit of smoking, the use of illicit drugs, hospital treatment and changing doctors, as well as the use of ART, were factors that affected the kinetics of CD4 counts over the follow-up period of patients living with HIV/AIDS. This study differs from previous research with the same object of study since it employs an appropriate methodology for longitudinal studies with repeated measures, considering three hierarchical levels. Repeated CD4 counts of patients (first level) were grouped on a higher level, which is the patient, with the obtained associations in the model on two levels, adjusted by a third level of aggregation formed by the groups of patients treated by the same doctor.

In the present study, patients aged 40 years and over presented lower rates of CD4 increase over the period of the cohort. Two lines of argument may be explored in order to explain this outcome: one is related to treatment adherence, and the other to immune senescence, a term used to describe phenotypic and functional alterations in lymphocytes associated with patient age. With regard to adherence, in the work of Lignani *et al.* [5] and Robbins *et al.* [13], patients aged 40 years and under were almost three times more likely to demonstrate poor adherence, thus suggesting a poorer response to treatment, however these studies did not evaluate the effects on CD4. This result runs counter to the findings of the present study. However, the purpose of those studies was not to assess the immune response by direct measurements of CD4, but by proxy. In a prospective cohort with a two-year follow-up, Hoffman *et al.* observed that patients aged 50 years and over presented a poor CD4 response to ART, thus corroborating the present findings, as well as a lower frequency of naive CD4 cells and higher number of memory CD4 cells in the pre-ART treatment assessment period, suggesting that the progression of HIV-infection is associated with a depreciation of naive CD4 cells and a lower response to ART [3]. This mechanism is also cited by NIES-Kaske, *et al.* [14], who indicated that an increase of central memory cells would be a manner, with which to compensate the deficit of naive cells. Florence *et al* [15] encountered a 50% increase in the likelihood of a low response in the CD4 count after initiating ART for each 10-year increase in patient age. Although they did not perform a qualitative assessment of the function of T-cells, one possible explanation put forward for the decrease in CD4, was that prolonged damage to the immune system of patients in an advanced stage of the disease may lead to cell dysfunction and/or a break in the increase of CD4 cells, thus reinforcing the hypothesis of immune senescence [14]. Other studies have corroborated a reduction in CD4 counts associated with increasing age [2], [16].

Among the variables related to patient habits, smoking and illicit drug use were discriminant factors for a lower increase of CD4 over time. However, these two variables did not remain in the same model, possibly because of the concomitance of the two habits. Considering the importance of each in the progression of the disease, two independent explanatory models were presented, to discuss the results. There is certain controversy with regard to smoking and declining levels of CD4. Some studies have corroborated the present findings, by describing lower levels of CD4 progression among smokers [16]–[19], others have shown no statistical significance for this association [20]–[24]. Some authors suggest that a drop in CD4 levels is associated to the fact that smokers living with HIV/AIDS have a greater susceptibility of developing respiratory diseases such as bacterial pneumonia, thus increasing their inflammatory levels, affecting the progression of the disease and increasing the risk of death [17]. Few studies have examined the relationship of treatment adherence with smoking. Shuter, *et al.*, in a cross-section of 64 patients, described the outcome of an association of smoking with poor adherence [25], however, Feldman *et al.* [18] concluded that there was no clear definition in the literature regarding the direct relationship between smoking and poor adherence to ART.

An association was observed between illicit drug use and the behavior of CD4 over time, with a lower rate of increase in CD4 counts among patients who used at least one illicit drug, thus corroborating the literature [16], [26]–[28]. Baum, *et al.* [26], estimated that in individuals who frequently used alcohol and crack, the risk of a decrease in CD4 counts was four times greater, regardless of whether they were on ART or not. Cook, et al., in a review article on the use of crack cocaine and progression of the disease in patients living with HIV, confirmed that cocaine is associated with immune changes in a variety of lymphocytes, including the natural killer cells, CD4 and CD8. They suggested, based on studies by Baldwin et al, that cocaine inhibits the effector function of neutrophils and macrophages, as well as reduces cytokine production, decreasing the immune response [27]. In two cohorts that aimed to assess the effect of marijuana on the immune response of patients living with HIV, there was no statistically significant association between the two variables [24], [29], in contrast to our findings in the univariate analysis. However, Chao, et al. concluded that their results did not exclude the possibility that drug use could negatively affect T-cell function, as previously mentioned [24]. The association between illicit drug use and adherence to ART treatment is cited by a number of authors [7], [30], but the relationship with adherence does not seem to be determinant in the decline of CD4, as in some studies with patients not on ART who use illicit drugs, the effect on CD4 decline over time was significant.

Two variables related to patient care remained in the final model of the behavior of CD4 over time: the referral health service where the patient is treated and a change of doctor during the follow-up period. With regard to the first, patients at Service “A” had a higher rate of CD4 increase over time than those treated at “B”. Differences in the clinical characteristics of the patients, or those related to the quality of care are possible explanations for this result. When comparing the profile of patients in the two referral services, it was observed that patients treated at Service “A” presented a lower mean time of HIV-infection diagnosis (5±2.6 years) when compared to those in Service “B” (6.4±3.8 years), as well as lower initial CD4 counts. Lower CD4 counts favour early therapeutic intervention resulting in a greater acceleration of increased CD4 counts in these patients. A longer period of infection decreases the patient's response to treatment. The proportion of patients using ART was similar in the two services. There was a higher percentage of patients who changed doctors during follow-up in Service “B” (11.1%) than in Service “A” (4.6%), which is one factor in this study that is associated with a lower rate of CD4 increase. Other features of the care provided for these patients were not focused upon in this study.

The aims of this study did not include the motives for changing doctors during follow-up, although this aspect constitutes a significant lacuna that needs to be explored in order to fully understand the quality of healthcare provided by health professionals. One of the factors that the World Health Organization indicates as being related to ART adherence is the patient/healthcare-provider relationship, where the object is the doctor-patient relationship. In a meta-analysis, a strong association was observed between doctor and patient communication and adherence to treatment, with the greatest risk of noncompliance (19%) among patients who reported having “poor” communication with their doctors [31]. Three qualitative studies that assessed the effect of the doctor-patient relationship [32]–[34], two with patients living with HIV, corroborate the hypothesis of impaired treatment adherence associated with the quality of the doctor-patient relationship. Although there is no data that allow us to conclude that the main factor for changing doctors was because of the relationship with the patient, it is important that other longitudinal studies are undertaken in order to assess factors specifically related to the care provided to patients living with HIV/AIDS.

The use of ART has an independent effect on the rate of CD4 increases over time. In the cohort of the present study, the mean CD4 increase in patients on ART was 1.155 cells/mm^3^ more when compared to patients not on ART, thus confirming the findings of the literature. According to Hoffman, et al. (2010) an appropriate response in CD4 counts of patients initiating ART is an annual growth of 50 to 150 cells/mm^3^. Considering the present cohort and the multivariate multilevel model proposed for patients on ART (Table 3), the mean monthly growth was estimated as 3.228 cells/mm^3^ per month, which corresponds to a mean annual growth of 38.74 cells/mm^3^, which is below WHO recommended treatment for an adequate response. This therefore, highlights the importance of further studies into the factors related to changes in CD4 and the adopted intervention strategies.

The study has some limitations. Patients were followed under routine conditions and data on CD4 count, viral load and antiretroviral treatment were obtained from medical records. Although patients were required by the attending physician to undertake a CD4 count and viral load determination every four months the time gap between consecutive exams varied among patients. Another point is that the information on these HIV related variables was available from the time of diagnosis; however information on the other variables was only available from enrollment in the cohort. Patients were selected from two different reference centres for the treatment of HIV/AIDS but exams were performed by the same reference laboratory and care is delivered in both services according to standard procedures, following the guidelines of the Brazilian Health Ministry for the whole country; thus the results are comparable.

The study pointed out groups that should be monitored more carefully because of their potential to a poorer response to treatment. However further studies will be very helpful to clarify some points. Other studies are necessary to understand to which extent smoking and illicit drug use affect the increase of CD4 through biological mechanisms or through an effect on adherence to antiretroviral treatment. The evidence that the quality of the care provided by health professionals may influence the response to the antiretroviral treatment is another aspect to be explored.
